# Correlation between whole skeleton dual energy CT calcium-subtracted attenuation and bone marrow infiltration in multiple myeloma

**DOI:** 10.1016/j.ejrad.2022.110223

**Published:** 2022-04

**Authors:** Renyang Gu, Ashik Amlani, Ulrike Haberland, Dan Hodson, Matthew Streetly, Michela Antonelli, Isabel Dregely, Vicky Goh, on behalf of the Myeloma Imaging Research Group at Guy's and St Thomas' Hospitals, London and King's College London

**Affiliations:** a Cancer Imaging, School of Biomedical Engineering and Imaging Sciences, King’s College London, St Thomas’ Hospital, Westminster Bridge Road, London SE1 7TH, United Kingdom; b Department of Radiology, Guy’s and St Thomas’ NHS Foundation Trust, London SE1 7TH, United Kingdom; c Siemens Healthineers, Siemensstrasse 1, 91301 Forchheim, Germany; d Department of Haematology and Oncology, Guy’s and St Thomas’ NHS Foundation Trust, London SE1 9RT, United Kingdom; e Biomedical Engineering, School of Biomedical Engineering and Imaging Sciences, King’s College London, St Thomas’ Hospital, Westminster Bridge Road, London, SE1 7TH London, United Kingdom

**Keywords:** Myeloma, Whole-body, Dual-energy CT, Image Segmentation, Calcium-subtracted attenuation, IMWG, International Myeloma Working Group

## Abstract

**Objectives:**

Objective evaluation of the extent of skeletal marrow involvement in multiple myeloma remains a clinical gap for CT. We aimed to develop a quantitative segmentation pipeline for dual energy CT and to assess whether quantified whole skeleton calcium-subtracted attenuation values correlate with biopsy-derived bone marrow infiltration in multiple myeloma.

**Methods:**

Consecutive prospective patients with suspected/established myeloma underwent dual source CT from the skull vertex to proximal tibia. Whole skeleton segmentation was performed for 120 kVp-equivalent images as follows: following Hounsfield unit (HU) thresholding, a Chan-Vese morphological operation was implemented to generate a whole skeleton segmentation mask. This mask was then applied to corresponding whole skeleton material decomposition calcium-subtracted maps, generating whole skeleton HU values. Associations with biopsy-derived bone marrow plasma cell infiltration percentage were assessed with Spearman’s rank correlation; significance was at 5%.

**Results:**

21 patients (12 females; median (IQR) 67 (61, 73) years) were included; 16 patients had osteolytic bone lesions; 15 patients underwent bone marrow biopsy. Segmentation and quantification were feasible in all patients. Median (IQR) of the average skeletal calcium-subtracted attenuation was −59.9 HU (-66.3, −51.8HU). There was a positive correlation with bone marrow plasma cell infiltration percentage (Spearman’s rho: + 0.79, *p < 0.001*).

**Conclusion:**

Whole skeleton calcium-subtracted attenuation is associated with the degree of bone marrow infiltration by plasma cells, providing an objective measure of marrow involvement with the potential to allow earlier detection of disease.

## Highlights

• Quantification of whole skeleton calcium-subtracted attenuation with dual energy CT is feasible.
• Whole skeleton calcium-subtracted attenuation correlates with the degree of marrow infiltration by plasma cells on bone marrow biopsy.
• Whole skeleton calcium-subtracted attenuation provides complementary information to the detection of osteolytic bone lesions.

## 1 Introduction

Multiple myeloma is a bone marrow haemopathy characterised by clonal plasma cells, causing pathological fractures, anaemia, recurrent infections, hypercalcaemia, and renal failure [1]. 140,000 new cases are diagnosed worldwide each year [2]. The 2019 International Myeloma Working Group (IMWG) imaging guidelines currently recommend whole body CT as a first line diagnostic test for suspected myeloma [3]. The presence of one or more osteolytic lesion on CT is a myeloma defining event [4]. However, false positive lesions may occur due to focal areas of yellow marrow, vertebral end plate degenerative changes or haemangiomas [5]. Marrow infiltration by plasma cells may be present prior to the development of visible osteolytic lesions on CT. Thus, assessment of skeletal marrow infiltration may detect disease or its progression at an earlier time point than current practice.

While CT attenuation assessment is not currently recommended for diagnosis by the IMWG guidelines, dual energy CT with reconstruction of calcium-subtracted attenuation maps offers an opportunity to quantify the degree of bone marrow infiltration. For example, a previous study of 53 patients with MRI of the axial skeleton as a reference standard showed differences in calcium-subtracted attenuation between normal, focal and diffuse imaging patterns with mean calcium attenuation (Hounsfield unit, HU) of −66HU, +3HU and −13HU, respectively [6].

To date, dual energy CT studies in myeloma patients have focussed on placing focal regions-of-interest within MRI correlated–CT bone lesions and/or within selected vertebrae or selected areas within the pelvis [6], [7], [8], [9]. Focal lesional attenuation at a high level of calcium suppression has also been shown to predict for 18F-FDG PET metabolic activity, with higher attenuation noted in metabolically active vs. non-active lesions [10]. More recently, the feasibility of artificial intelligence tools to segment the thoracolumbar spine and quantify attenuation have been explored [11]. Here, quantified CT of the non-fatty portion of spinal bone marrow predicted pelvic bone marrow infiltration after adjustment for bone mineral density with r = 0.46 [11]. However, tools for objective assessment of the whole skeleton are still lacking.

We hypothesized that dual energy CT derived calcium-subtracted attenuation of the whole imaged skeleton will reflect the degree of bone marrow infiltration, providing complementary information to the presence of focal osteolytic lesions or regional marrow assessment.

Thus, we aimed to assess the feasibility of whole skeleton quantification of calcium-subtracted attenuation; and to assess its correlation with bone marrow plasma cell infiltration percentage from biopsy. Secondarily, we aimed to correlate whole skeleton calcium-subtracted HU attenuation values with other clinical variables (haemoglobin level and age); and to compare whole skeleton calcium-subtracted HU attenuation values to those derived from focal marrow evaluation and osteolytic lesions.

## 2 Material and methods

Institutional approval was obtained for this study and patients provided informed consent for this analysis.

### 2.1 Patients

**Fig. 1:**
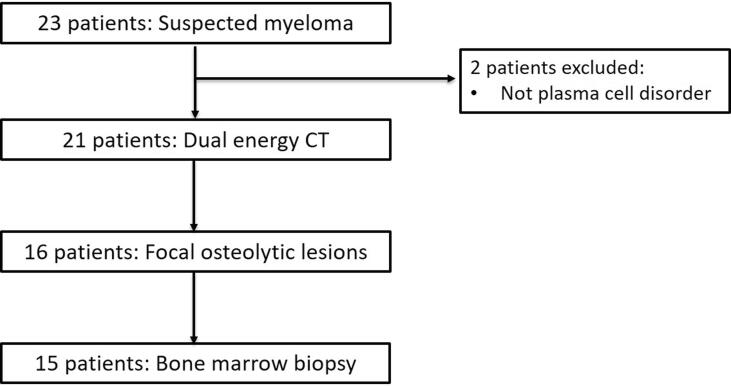
Study flowchart.

Consecutive patients with suspected or proven newly-diagnosed or relapsed myeloma, who were unable to undergo whole body MRI as per our usual clinical practice (for reasons including claustrophobia (n = 5); whole body MRI not tolerated due to symptoms (n = 5); non-MRI compatible implant (n = 1); physician triage/choice (n = 10)) prior to treatment, and who attended for a dual energy CT between 01 February 2018 and 01 April 2021 were included. There were no predefined exclusions. The study flowchart is summarised in Fig. 1**.**

### 2.2 Dual energy CT acquisition, image reconstruction and post-processing

All patients underwent a supine non-contrast dual energy CT from the skull vertex to proximal tibia on a third generation dual source CT scanner (Somatom Force, Siemens Healthineers, Forchheim, Germany) with the following acquisition parameters: tube voltages 90 kV (A) / 150 kV with tin filter (B); collimation, 128x 0.6 mm; pitch, 0.6; rotation time, 0.5 s; automated attenuation based tube current modulation (Care dose 4D; Siemens Healthineers, Forchheim, Germany); and quality reference tube current- time product, 1.6:1.

Axial images (slice thickness 1.0 mm; 0.75 mm increment; 500 mm field-of-view; soft tissue kernel QR40; advanced model iterative reconstruction (ADMIRE) level 3) were reconstructed for 90kVp and 150kVp datasets. Axial weighted-average images with equivalent contrast to single energy 120kVp images were also derived from these datasets with a 50:50 mixing ratio for viewing purposes as per our clinical practice.

Virtual calcium-subtracted CT images were calculated with a three-material decomposition algorithm for bone mineral, yellow marrow, and red marrow on dedicated software (Syngo.via VB30; Siemens Healthineers, Forchheim, Germany) using default settings: calcium slope, 1.65; yellow marrow, −108/-84 HU (90/150kVp); red marrow, 52/51HU (90/150kVp).

**Fig. 2:**
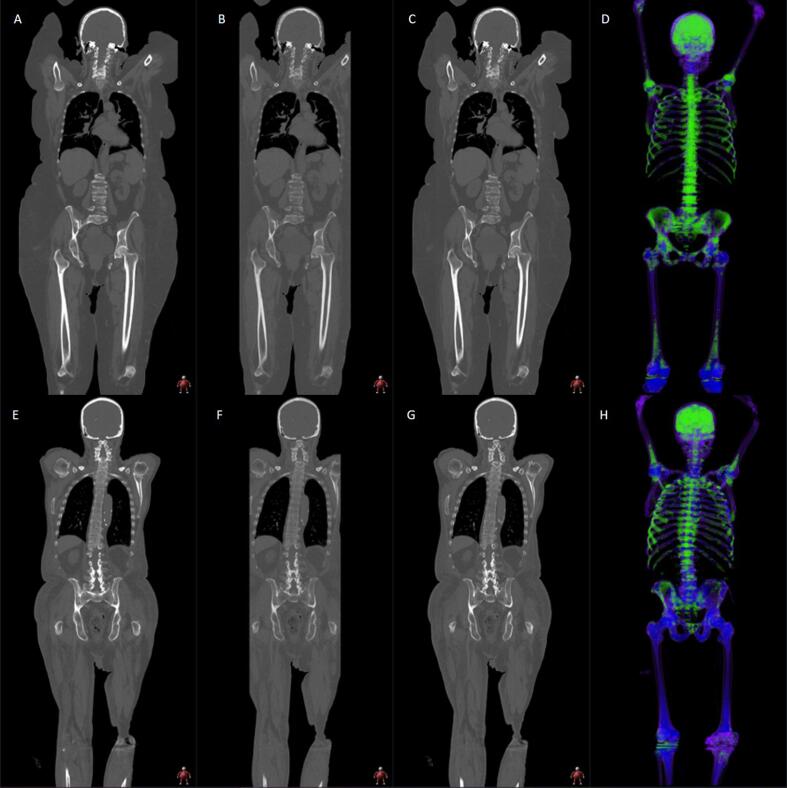
60-year old female with a new diagnosis of myeloma (top row). Representative dual-energy CT images show multifocal disease and relatively diffuse marrow infiltration, particularly within the axial skeleton. Reconstructed coronal 90kVp (A); 150 kVp (B); weighted-average 120 kVp images (C); and colour-coded whole body rendered image (green representing infiltration) (D). 88-year old male with a new diagnosis of myeloma (bottom row). Representative dual-energy CT images show multifocal disease but patchy heterogeneous marrow infiltration. Reconstructed coronal 90kVp (E); 150 kVp (F); weighted-average 120 kVp images (G); and colour-coded whole body rendered image (green representing infiltration) (H).

For viewing purposes, dual energy CT images were also displayed as weighted-average CT images with a colour-coded virtual calcium-subtracted CT overlay using the bone marrow settings. Representative patient examples are shown in Fig. 2. The mean ± SD CT dose index was 7.81 ± 1.96 mGy.cm and the mean ± SD dose-length product was 1017 ± 322 mGy.cm for this cohort.

### 2.3 Segmentation and generation of whole skeleton calcium-subtracted attenuation values

**Fig. 3:**
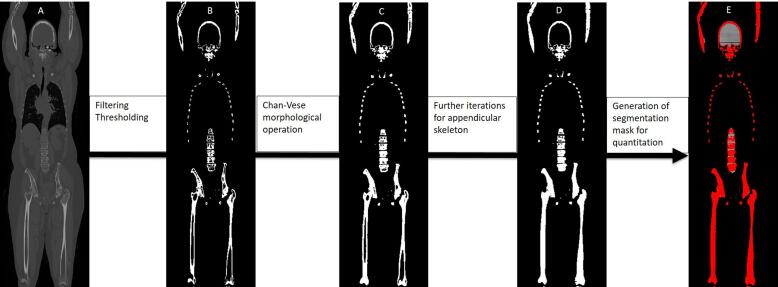
Schematic showing workflow for whole skeleton segmentation and quantification. A: Coronal weighted-average 120 kVp image (bone windows); B: Initial segmentation with filtering and thresholding at 120HU; C: Segmentation following 2 iterations of the Chan-Vese morphological operation; D: Segmentation following further iterations to complete delineation and filling in (erosion) of the appendicular skeleton; E: Final segmentation mask overlaid on calcium-subtracted attenuation parametric map to generate whole skeleton histogram HU values.

Anonymised images were exported for further post-processing. Whole skeleton segmentation and quantification was undertaken using an in-house developed semi-automatic pipeline written in Python within the Pytorch environment. The pipeline is illustrated in Fig. 3. The weighted-average 120 kVp equivalent CT images were reconstructed with bone windows (W: 1800 L:400) and normalised with the median HU value. Following stepwise assessment for the optimal thresholding value above 100HU for contouring the skeleton, a fixed thresholding value of 120 HU was then applied to generate an initial contour for further post-processing. Next, to fill in this contour and to exclude non-skeletal structures (i.e. brain, spinal cord, spinal canal), a Chan-Vese morphological operation (morphological active contours without edges) was applied [12].

The Chan-Vese morphological operation iteratively minimises the energy function *F*(*c*_1_,*c*_2_,*C*) by the following equation:

$${\mathrm{argmin}}_{c_{1},c_{2},C}\mu \cdot Length\left(C\right)+\lambda_{1}\cdot \underset{inside\left(C\right)}{\int^{\;}}\left|\left(x,y\right)-c_{1}{|}^{2}dxdy+\lambda_{2}\cdot \underset{outside\left(C\right)}{\int^{\;}}\right|\left(x,y\right)-c_{2}{|}^{2}dxdy$$

where, *µ* = 1 is the penalty on the total length of the boundary of the segmented region (*C*); while *λ*_1_,*λ*_2_ are two weighting parameters affecting the uniformity of the inner contour (*inside*(*C*)) and outer contour region (*outside*(*C*)). Here, both were set to 1 for binary mask generation (i.e., c_1_ = c_2_ = 1).

With this operation, the brain, spinal cord, spinal canal, and cerebrospinal fluid were excluded as the mean pixel value of the inner contour region is substantially different from the outer contour region. Two iterations of the Chan-Vese morphological operation were used initially for the whole skeleton. The mask was then reviewed by a radiologist, and, where necessary, further slice numbers were inputted to enable further filling in (‘erosion’) of the skeleton of selected images (e.g., of the femur or humerus) to ensure that the skeletal bone marrow mask was complete and representative. Any non-skeletal structures included in the process, e.g. hip prosthesis, were removed by further HU thresholding ± cropping. The resultant skeletal bone marrow mask was then applied directly to the corresponding calcium-subtracted attenuation parametric map to extract a histogram of bone marrow HU values for the whole skeleton.

### 2.4 Generation of regional marrow and lesion calcium-subtracted attenuation values

In all patients, using the vendor dedicated dual energy CT software on Syngo.via (VB30; Siemens Healthineers, Forchheim, Germany) a standard circular region-of-interest was placed within the L3 vertebra and right iliac bone (excluding any focal osteolytic lesion, if present) to generate calcium-subtracted attenuation values. Mean and standard deviation calcium-subtracted Hounsfield unit values were generated by the software and recorded.

In patients with focal osteolytic bone lesions, up to 5 focal skeletal lesions (>5mm) were identified on the weighted-average images and corresponding calcium-subtracted images. A circular region-of-interest was placed within the boundaries of each lesion. Again, mean and standard deviation calcium-subtracted Hounsfield unit values for each lesion generated by the software were recorded.

### 2.5 Clinical management and follow up

This was undertaken as per usual institutional practice. Patients were staged using the International Staging System. Bone marrow biopsy was performed where this directed further clinical management; sampling was from the iliac crest. Blood tests included a full blood count, urea and electrolytes, and bone biochemistry.

### 2.6 Statistical analysis

Descriptive statistics were performed for patient and disease characteristics. Spearman’s rank correlation assessed associations between whole skeleton calcium-subtracted attenuation and biopsy derived marrow plasma cell infiltration level, blood haemoglobin level, and age, respectively. Wilcoxon signed-rank test was performed to compare grouped lesion calcium-subtracted attenuation, grouped regional and whole skeleton calcium-subtracted attenuation.

## 3 Results

### 3.1 Patients

21 patients (12 females, 9 males; median (IQR) age 67 (61,73) years) were included in this analysis. The majority (13/21, 62%) presented with relapsed myeloma. 16/21 (76%) patients had focal osteolytic lesions. 15/21 (71%) patients underwent subsequent bone marrow biopsy, all with a new myeloma diagnosis or newly relapsed disease. Patient and disease characteristics are summarised in Table 1.

**Table 1:** Patient and disease characteristics.

**Characteristics**		**Number (%)**
**Gender**	Male	9 (43)
	Female	12 (57)
**Diagnosis**	Monoclonal gammopathy of unknown significance	3 (14)
	New diagnosis myeloma	5 (24)
	Relapsed myeloma	13 (62)
**Subtypes**	IgG kappa	12 (57)
	IgG lambda	2 (10)
	Lambda light chain	4 (19)
	IgA	1 (5)
	Missing data	2 (10)
**International Staging System**	Stage I	6 (29)
	Stage II	4 (19)
	Stage III	4 (19)
	Missing data	7 (33)
**Haemoglobin level**	Normal range (120–150 g/L)	9 (43)
	Below normal range (<120 g/L)	12 (57)
**Creatinine level**	Normal range (48–84 μmol/L)	8 (38)
	Above normal range (>84 μmol/L)	11 (52)
	Below normal range (<48 μmol/L)	2 (10)
**Calcium level**	Normal range (2.15–2.55 mmol/L)	15 (71)
	Above normal range(>2.55 mmol/L)	1 (5)
	Below normal range(<2.15 mmol/L)	4 (19)
**Bone marrow biopsy: plasma cell infiltration percentage**	10%	3 (14)
	11–60%	6 (29)
	Above 60%	6 (29)
	Missing data*	6 (29)
**Outcome**	Alive	13 (62)
	Deceased	8 (38)
* not clinically indicated (n = 3, monoclonal gammopathy); deceased (n = 2); not performed (n = 1), all with relapsed myeloma.		

### 3.2 Whole skeleton segmentation and calcium-subtracted attenuation

Segmentation was feasible in all patients. In 17/21 (81%) patients, no further adjustments were required to the skeletal marrow mask generated by the segmentation pipeline. In 3/21 (14%) patients, metal prostheses and a loop of hyperdense bowel required additional post-processing for removal. Image processing time ranged from 5 to 8 min, depending on the need for additional adjustments. Histogram values derived from whole skeleton segmentation for each patient are summarised in Table 2**.**

**Table 2:** Histogram values derived from whole skeleton segmentation for each patient.

**Patient ID**	**Diagnosis**	**Bone Marrow Calcium Subtracted Attenuation (Hounsfield Unit, HU)**				
		**Mean**	**Median**	**SD**	**Skewness**	**Kurtosis**
01	Relapsed myeloma	−63.9	−72.0	47.8	1.2	4.6
02	Relapsed myeloma	−59.2	−64.0	45.4	1.6	26.8
03	Relapsed myeloma	−63.6	−77.0	100.0	13.9	315.2
04	New myeloma	−39.1	−35.0	47.5	1.1	17.8
05	Relapsed myeloma	−54.3	−58.0	45.3	0.5	0.3
06	Relapsed myeloma	−47.4	−50.0	54.6	0.6	1.4
07	New myeloma	−82.5	−91.0	49.4	1.0	3.4
08	Relapsed myeloma	−53.9	−55.0	46.6	1.1	7.7
09	Relapsed myeloma	−53.3	−55.0	36.4	0.9	8.4
10	Relapsed myeloma	−64.6	−69.0	45.4	1.3	5.3
11	New myeloma	−26.1	−28.0	96.7	7.0	87.2
12	Relapsed myeloma	−25.5	−25.0	71.2	2.7	25.1
13	MGUS – light chain	−58.1	−79.0	108.8	5.0	31.1
14	Relapsed myeloma	−41.9	−46.0	80.7	7.6	96.2
15	MGUS - IgA	−72.2	−79.0	51.8	1.6	10.6
16	New myeloma	−62.3	−83.0	143.3	9.3	121.6
17	Relapsed myeloma	−75.8	−83.0	58.0	2.8	26.4
18	MGUS – IgG kappa	−79.7	−82.0	44.4	0.2	0.4
19	Relapsed myeloma	−59.9	−63.0	49.4	3.5	69.2
20	New Myeloma	−66.3	−84.0	81.3	9.8	224.4
21	New Myeloma	−77.5	−83.0	49.2	2.4	28.2

Abbreviation: MGUS = monoclonal gammopathy of unknown significance; SD = standard deviation

**Fig. 4:**
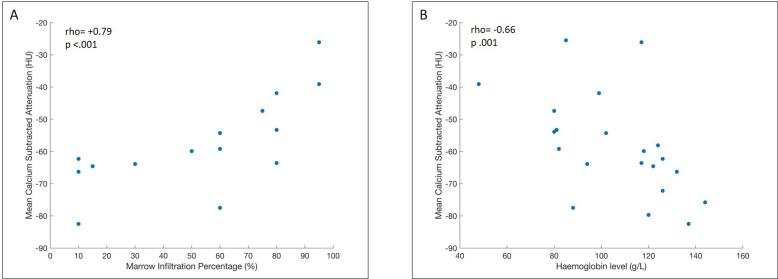
Correlation plots showing positive association of whole skeleton calcium-subtracted attenuation values with bone marrow biopsy (A); and negative association of whole skeleton calcium-subtracted attenuation values with haemoglobin level (B). All patients had new or relapsed myeloma with biopsy infiltration ≥ 10%).

Median (IQR) of average whole skeleton calcium-subtracted attenuation was −59.9 HU (-66.3, −51.8HU). There was a strong positive correlation with biopsy-derived bone marrow plasma cell infiltration percentage (Spearman’s rho + 0.79, p < 0.001) and a negative correlation with haemoglobin level (Spearman’s rho −0.66, p = 0.001) (Fig. 4). There was no correlation between whole skeleton calcium-subtracted attenuation and age (Spearman’s rho −0.22, p = 0.34).

### 3.3 Comparison of whole skeleton with regional marrow calcium-subtracted attenuation values

Compared with regional average calcium-subtracted attenuation derived from L3 and the iliac crest, whole skeleton average calcium-subtracted attenuation had a lower median value (-59.9 HU vs. −51.3 HU; p < 0.001) highlighting regional differences in marrow values.

### 3.4 Comparison of whole skeleton with lesion calcium-subtracted attenuation values

Compared with lesion average calcium-subtracted attenuation, whole skeleton average calcium-subtracted attenuation had a lower median value (-56.8 HU vs. −11.8 HU; p < 0.001) indicating the complementary nature of these measurements.

## 4 Discussion

There is still an outstanding clinical need for earlier and more objective assessment of skeletal infiltration by plasma cells in multiple myeloma. In addition to osteolytic lesions arising from bone destruction, osteoporosis has been reported in patients with multiple myeloma undergoing CT [13] indicating a more diffuse process underlying CT findings. To date, assessment in clinical practice has been limited by the lack of whole skeleton assessment tools, with published studies reporting on regional analysis from focal region-of-interest analysis of the lumbar spine or pelvis.

In this cohort of patients, we found that whole skeleton segmentation was feasible using an in-house segmentation and quantification pipeline, and that whole skeleton calcium-subtracted attenuation values correlated positively with biopsy plasma cell infiltration level, yet, were distinct from regional marrow and lesional calcium-subtracted attenuation values, indicating their complementary nature for diagnosis and ongoing disease assessment. The processing time of 5–8 min was acceptable for clinical practice. Further adjustment of the segmentation mask was required in 10% of patients in our cohort, predominantly to remove metal prostheses. An advantage of this pipeline is that it would also be applicable to other dual energy CT methods including those integrated into PET/CT scanners and with calcium-subtracted mapping capabilities.

Previous dual energy CT studies have indicated that visual analysis may detect bone marrow infiltration with good diagnostic performance, but this will always be subject to observer variation. Calcium-subtracted cut-off values of −44.9HU have been quoted for the presence of bone marrow infiltration (using MRI as reference standard) with a sensitivity and specificity of 93% [7]. However, being derived from focal region-of-interest analysis rather than the whole skeleton, such analysis may not capture the heterogenous distribution of plasma cells associated with myeloma throughout the skeleton. Similarly, cut-off values for lytic lesions of −3 HU on CT have been quoted [8]. Our quantitative values were in a comparable range to these previously quoted values but clearly, whole skeleton analysis provides more objective evidence of the global extent of marrow infiltration.

To date, studies have highlighted a role for MRI rather than CT for the evaluation of marrow infiltration in multiple myeloma due to MRI’s higher sensitivity for infiltrative disease compared to standard CT [14]. In addition to the assessment of different patterns of bone marrow involvement (normal, focal, homogeneous diffuse infiltration, combined focal and diffuse infiltration and variegated or ‘salt and pepper’) [15], quantitative assessment of skeletal T1-weighted gradient echo Dixon derived fat-signal fraction and diffusion-weighted MRI derived apparent diffusion co-efficient have advocated e.g., of focal lesions for therapy assessment in clinical trial settings [16], [17], [18], [19]. In terms of marrow assessment, fat-signal fraction within the spine (L1-L3) has been shown to be lower in symptomatic versus asymptomatic myeloma [20], [21]; and lower in symptomatic versus healthy controls [22], assumed to indicate replacement of marrow fat cells by plasma cell infiltration. Conversely, apparent diffusion co-efficient derived from ROI analysis of the thoracic and lumbar spine have been shown to be higher in myeloma patients with diffuse infiltration compared to asymptomatic myeloma patients and healthy controls [22]. As with quantitative dual energy CT and 18F-FDG PET, a challenge remains of differentiation of infiltrative disease from hyperplastic haematopoietic bone marrow, with lower therapeutic assessment performance in patients with anaemia [23]. Combining assessment of fat-signal fraction and apparent diffusion coefficient with anatomical appearances may potentially mitigate this [24].

For patients who cannot undergo whole body MRI, dual energy CT provides an alternative. Nevertheless, there are limitations to this study. First, the sample size for this feasibility study was small. However, the findings remain encouraging. Second, although there was a positive correlation with plasma cell infiltration, a number of additional factors are known to contribute to skeletal calcium-subtracted attenuation values including the level of other marrow components, e.g., haematopoietic cells; this was suggested indirectly by the negative correlation with haemoglobin level in our study. Third, although we employed a phantom-optimized dual energy CT protocol balancing radiation dose to sufficient image quality for accurate quantification, this cannot be deemed a low-dose CT protocol. Thus, the benefits of quantification have to be balanced against a radiation burden to the patient. Further exploration of lower dose techniques including low dose dual energy CT with higher levels of modelled iterative reconstruction or dual layer detector dual energy CT is required. Fourth, our cohort were preselected, and underwent dual energy CT rather than whole body MRI thus no comparisons could be undertaken between both modalities in this cohort. Finally, day-to-day test-retest analysis was not performed in this cohort as only one CT examination was performed but would be valuable information to obtain in the future.

In conclusion, whole skeleton segmentation was feasible. Calcium-subtracted attenuation was associated positively with the degree of marrow infiltration, differed from lesional values, and may provide a more objective measure of overall disease burden than current practice.

## References

[1] Rajkumar S.V. (2020). Multiple myeloma: 2020 update on diagnosis, risk-stratification and management. Am. J. Hematol..

[2] Cowan A.J., Allen C., Barac A., Basaleem H., Bensenor I., Curado M.P., Foreman K., Gupta R., Harvey J., Hosgood H.D., Jakovljevic M., Khader Y., Linn S., Lad D., Mantovani L., Nong V.M., Mokdad A., Naghavi M., Postma M., Roshandel G., Shackelford K., Sisay M., Nguyen C.T., Tran T.T., Xuan B.T., Ukwaja K.N., Vollset S.E., Weiderpass E., Libby E.N., Fitzmaurice C. (2018). Global Burden of Multiple Myeloma: A Systematic Analysis for the Global Burden of Disease Study 2016. JAMA Oncol.

[3] Hillengass J., Usmani S., Rajkumar S.V., Durie B.G.M., Mateos M.-V., Lonial S., Joao C., Anderson K.C., García-Sanz R., Riva E., Du J., van de Donk N., Berdeja J.G., Terpos E., Zamagni E., Kyle R.A., San Miguel J., Goldschmidt H., Giralt S., Kumar S., Raje N., Ludwig H., Ocio E., Schots R., Einsele H., Schjesvold F., Chen W.-M., Abildgaard N., Lipe B.C., Dytfeld D., Wirk B.M., Drake M., Cavo M., Lahuerta J.J., Lentzsch S. (2019). International myeloma working group consensus recommendations on imaging in monoclonal plasma cell disorders. Lancet Oncol..

[4] Rajkumar S.V., Dimopoulos M.A., Palumbo A., Blade J., Merlini G., Mateos M.-V., Kumar S., Hillengass J., Kastritis E., Richardson P., Landgren O., Paiva B., Dispenzieri A., Weiss B., LeLeu X., Zweegman S., Lonial S., Rosinol L., Zamagni E., Jagannath S., Sezer O., Kristinsson S.Y., Caers J.o., Usmani S.Z., Lahuerta J.J., Johnsen H.E., Beksac M., Cavo M., Goldschmidt H., Terpos E., Kyle R.A., Anderson K.C., Durie B.G.M., Miguel J.F.S. (2014). International Myeloma Working Group updated criteria for the diagnosis of multiple myeloma. Lancet Oncol..

[5] Treitl K.M., Ricke J., Baur-Melnyk A. (2022). Whole-body magnetic resonance imaging (WBMRI) versus whole-body computed tomography (WBCT) for myeloma imaging and staging. Skeletal Radiol..

[6] Kosmala A., Weng A.M., Krauss B., Knop S., Bley T.A., Petritsch B. (2018). Dual-energy CT of the bone marrow in multiple myeloma: diagnostic accuracy for quantitative differentiation of infiltration patterns. Eur. Radiol..

[7] Kosmala A., Weng A.M., Heidemeier A., Krauss B., Knop S., Bley T.A., Petritsch B. (2018). Multiple Myeloma and Dual-Energy CT: Diagnostic Accuracy of Virtual Noncalcium Technique for Detection of Bone Marrow Infiltration of the Spine and Pelvis. Radiology.

[8] Thomas C., Schabel C., Krauss B., Weisel K., Bongers M., Claussen C.D., Horger M. (2015). Dual-energy CT: virtual calcium subtraction for assessment of bone marrow involvement of the spine in multiple myeloma. AJR Am. J. Roentgenol..

[9] Reinert C.P., Krieg E., Esser M., Nikolaou K., Bösmüller H., Horger M. (2021). Role of computed tomography texture analysis using dual-energy-based bone marrow imaging for multiple myeloma characterization: comparison with histology and established serologic parameters. Eur. Radiol..

[10] Fervers P., Glauner A., Gertz R., Täger P., Kottlors J., Maintz D., Borggrefe J. (2021). Virtual calcium-suppression in dual energy computed tomography predicts metabolic activity of focal MM lesions as determined by fluorodeoxyglucose positron-emission-tomography. Eur. J. Radiol..

[11] Fervers P., Fervers F., Kottlors J., Lohneis P., Pollman-Schweckhorst P., Zaytoun H., Rinneburger M., Maintz D., Große Hokamp N. (2021). Feasibility of artificial intelligence-supported assessment of bone marrow infiltration using dual-energy computed tomography in patients with evidence of monoclonal protein - a retrospective observational study. Eur. Radiol..

[12] Chan T.F., Vese L.A. (2001). Active contours without edges. IEEE Trans. Image Process..

[13] J. Hillengass L.A. Moulopoulos S. Delorme V. Koutoulidis J. Mosebach T. Hielscher M. Drake S.V. Rajkumar B. Oestergaard N. Abildgaard M. Hinge T. Plesner Y. Suehara K. Matsue N. Withofs J. Caers A. Waage H. Goldschmidt M.A. Dimopoulos S. Lentzsch B. Durie E. Terpos Whole-body computed tomography versus conventional skeletal survey in patients with multiple myeloma: a study of the International Myeloma Working Group Blood cancer journal 7 (8) (2017) e599. 10.1038/bcj.2017.78 PMC5596388 28841211

[14] Dimopoulos M.A., Hillengass J., Usmani S., Zamagni E., Lentzsch S., Davies F.E., Raje N., Sezer O., Zweegman S., Shah J., Badros A., Shimizu K., Moreau P., Chim C.S., Lahuerta J.J., Hou J., Jurczyszyn A., Goldschmidt H., Sonneveld P., Palumbo A., Ludwig H., Cavo M., Barlogie B., Anderson K., Roodman G.D., Rajkumar S.V., Durie B.G., Terpos E. (2015). Role of magnetic resonance imaging in the management of patients with multiple myeloma: a consensus statement. J. Clin. Oncol.: official J. Am. Soc. Clin. Oncol..

[15] Hillengass J., Landgren O. (2013). Challenges and opportunities of novel imaging techniques in monoclonal plasma cell disorders: imaging “early myeloma”. Leukemia lymphoma.

[16] Messiou C., Hillengass J., Delorme S., Lecouvet F.E., Moulopoulos L.A., Collins D.J., Blackledge M.D., Abildgaard N., Østergaard B., Schlemmer H.P., Landgren O., Asmussen J.T., Kaiser M.F., Padhani A. (2019). Guidelines for Acquisition, Interpretation, and Reporting of Whole-Body MRI in Myeloma: Myeloma Response Assessment and Diagnosis System (MY-RADS). Radiology.

[17] Messiou C., Giles S., Collins D.J., West S., Davies F.E., Morgan G.J., Desouza N.M. (2012). Assessing response of myeloma bone disease with diffusion-weighted MRI. Brit. J. Radiol..

[18] Latifoltojar A., Hall-Craggs M., Rabin N., Popat R., Bainbridge A., Dikaios N., Sokolska M., Rismani A., D'Sa S., Punwani S., Yong K. (2017). Whole body magnetic resonance imaging in newly diagnosed multiple myeloma: early changes in lesional signal fat fraction predict disease response. Br. J. Haematol..

[19] Latifoltojar A., Hall-Craggs M., Bainbridge A., Rabin N., Popat R., Rismani A., D’Sa S., Dikaios N., Sokolska M., Antonelli M., Ourselin S., Yong K., Taylor S.A., Halligan S., Punwani S. (2017). Whole-body MRI quantitative biomarkers are associated significantly with treatment response in patients with newly diagnosed symptomatic multiple myeloma following bortezomib induction. Eur. Radiol..

[20] Takasu M., Kaichi Y., Tani C., Date S., Akiyama Y., Kuroda Y., Sakai A., Awai K., Gelovani J.G. (2015). Iterative decomposition of water and fat with echo asymmetry and least-squares estimation (IDEAL) magnetic resonance imaging as a biomarker for symptomatic multiple myeloma. PLoS ONE.

[21] Takasu M., Tani C., Sakoda Y., Ishikawa M., Tanitame K., Date S., Akiyama Y., Sakai A., Asaoku H., Kajima T., Awai K. (2012). Iterative decomposition of water and fat with echo asymmetry and least-squares estimation (IDEAL) imaging of multiple myeloma: initial clinical efficiency results. Eur. Radiol..

[22] Berardo S., Sukhovei L., Andorno S., Carriero A., Stecco A. (2021). Quantitative bone marrow magnetic resonance imaging through apparent diffusion coefficient and fat fraction in multiple myeloma patients. Radiol. Med. (Torino).

[23] Dong H., Huang W., Ji X., Huang L., Zou D., Hao M., Deng S., Shen Z., Lu X., Wang J., Song Z., Zhang X., Xue H., Xia S. (2021). Prediction of Early Treatment Response in Multiple Myeloma Using MY-RADS Total Burden Score, ADC, and Fat Fraction From Whole-Body MRI: Impact of Anemia on Predictive Performance. AJR Am. J. Roentgenol..

[24] Sun M., Cheng J., Ren C., Zhang Y., Li Y., Wang L., Liu Y. (2021). Differentiation of Diffuse Infiltration Pattern in Multiple Myeloma From Hyperplastic Hematopoietic Bone Marrow: Qualitative and Quantitative Analysis Using Whole-Body MRI. J. Mag. Resonance Imag.: JMRI.

